# *N*-(6-{2-[6-(2,2-Dimethyl­propanamido)-2-pyrid­yl]eth­yl}-2-pyrid­yl)-2,2-dimethyl­propanamide

**DOI:** 10.1107/S1600536810023068

**Published:** 2010-07-10

**Authors:** Hoong-Kun Fun, Wan-Sin Loh, Nirmal Kumar Das, Debabrata Sen, Shyamaprosad Goswami

**Affiliations:** a X-ray Crystallography Unit, School of Physics, Universiti Sains Malaysia, 11800 USM, Penang, Malaysia; b Department of Chemistry, Bengal Engineering and Science University, Shibpur, Howrah 711 103, West Bengal, India

## Abstract

The title compound, C_22_H_30_N_4_O_2_, lies about a crystallographic inversion center. The whole mol­ecule is disordered over two positions with a refined occupancy ratio of 0.636 (10):0.364 (10). The pyridine rings are approximately planar, with maximum deviations of 0.033 (10) and 0.063 (17) Å for the major and minor components, respectively. The mean planes of the pyridine rings form dihedral angles of 17 (2)° in the major component and 18 (2)° in the minor component with the respective formamide groups attached to them. In the crystal packing, inter­molecular N—H⋯O and C—H⋯O hydrogen bonds link the mol­ecules into two-dimensional networks parallel to the *ab* plane.

## Related literature

For the importance of dicarb­oxy­lic acids and their derivatives, see: Garcia-Tellado *et al.* (1990 ▶); Geib *et al.* (1993 ▶); Karle *et al.* (1997 ▶); Goswami, Dey, Fun *et al.* (2005 ▶); Goswami *et al.* (2006 ▶, 2008 ▶). For a related structure, see: Goswami, Dey, Chantra­promma *et al.* (2005 ▶). For the preparation, see: Yamada & Momose (1981 ▶); Goswami *et al.* (1989 ▶).
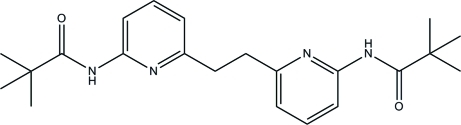


## Experimental

### 

#### Crystal data

C_22_H_30_N_4_O_2_
*M* *_r_* = 382.50
Orthorhombic, 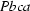

*a* = 11.7933 (3) Å
*b* = 10.3648 (2) Å
*c* = 17.8667 (4) Å
*V* = 2183.94 (9) Å^3^
*Z* = 4
Mo *K*α radiation
μ = 0.08 mm^−1^
*T* = 296 K
0.36 × 0.15 × 0.10 mm

#### Data collection

Bruker SMART APEXII CCD area-detector diffractometer
Absorption correction: multi-scan (*SADABS*; Bruker, 2009 ▶) *T*_min_ = 0.973, *T*_max_ = 0.992
36712 measured reflections
3221 independent reflections
1678 reflections with *I* > 2σ(*I*)
*R*_int_ = 0.076

#### Refinement

*R*[*F* ^2^ > 2σ(*F* ^2^)] = 0.062
*wR*(*F* ^2^) = 0.161
*S* = 1.03
3221 reflections
256 parameters
12 restraints
H-atom parameters constrained
Δρ_max_ = 0.17 e Å^−3^
Δρ_min_ = −0.16 e Å^−3^

### 

Data collection: *APEX2* (Bruker, 2009 ▶); cell refinement: *SAINT* (Bruker, 2009 ▶); data reduction: *SAINT*; program(s) used to solve structure: *SHELXTL* (Sheldrick, 2008 ▶); program(s) used to refine structure: *SHELXTL*; molecular graphics: *SHELXTL*; software used to prepare material for publication: *SHELXTL* and *PLATON* (Spek, 2009 ▶).

## Supplementary Material

Crystal structure: contains datablocks global, I. DOI: 10.1107/S1600536810023068/sj5021sup1.cif

Structure factors: contains datablocks I. DOI: 10.1107/S1600536810023068/sj5021Isup2.hkl

Additional supplementary materials:  crystallographic information; 3D view; checkCIF report

## Figures and Tables

**Table 1:** Hydrogen-bond geometry (Å, °)

*D*—H⋯*A*	*D*—H	H⋯*A*	*D*⋯*A*	*D*—H⋯*A*
C10*A*—H10*C*⋯O1*A*^i^	0.96	2.46	3.409 (12)	171
N2*A*—H2*AB*⋯O1*A*^i^	0.86	2.26	3.100 (16)	168

Symmetry code: (i) 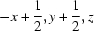.

**Fig. 1.**
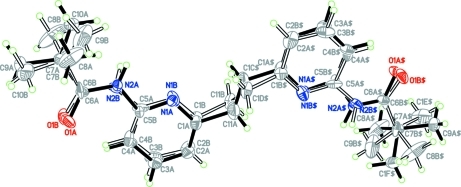
The molecular structure of the title compound, showing 20% probability displacement ellipsoids and the atom-numbering scheme. Both major and minor components are shown. Atoms with suffix $ are generated by the symmetry code -x, -y + 1, -z + 1.

**Fig. 2.**
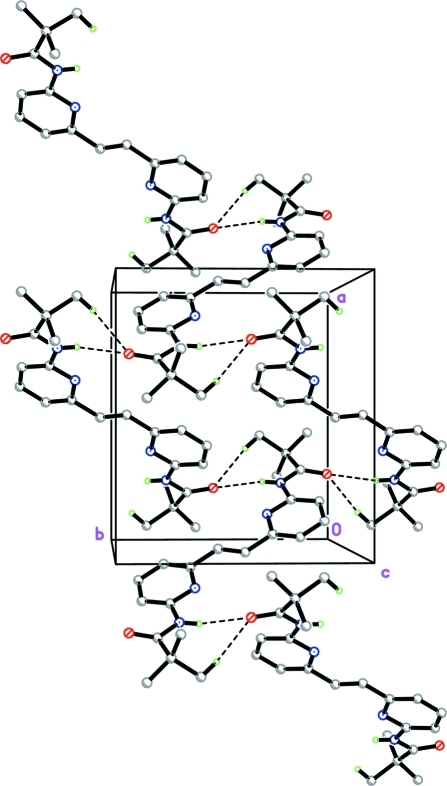
The two-dimensional networks formed by intermolecular N—H···O and C—H···O hydrogen bonds (dashed lines) parallel to the ab plane. H atoms not involved in intermolecular interactions (dashed lines) have been omitted for clarity. Only the major disorder component is shown.

**Fig. 3.**
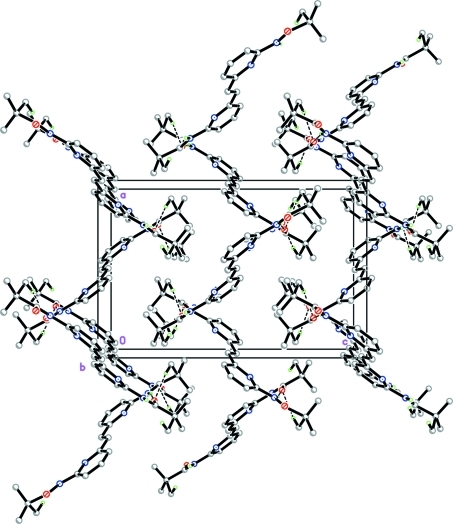
The crystal packing of the title compound, viewed along the b axis, showing the two-dimensional networks. H atoms not involved in intermolecular interactions have been omitted for clarity. Only the major disorder component is shown.

## supplementary crystallographic information

### Comment

The recognition of biologically important substrates like dicarboxylic acids by bis-pyridine amide is one of the most important areas of research in supramolecular chemistry as well as in the design of materials through new crystal engineering (Garcia-Tellado *et al.*, 1990; Geib *et al.*, 1993; Karle *et al.*, 1997; Goswami, Dey, Fun *et al.*, 2005; Goswami *et al.*, 2006, 2008). The title compound can be used as receptor for dicarboxylic acids with the ethylene group acting as a spacer.

The title compound, (Fig. 1), lies about a crystallographic inversion center (symmetry code = -*x*, -*y* + 1, -*z* + 1). The molecule has a whole-molecule disorder over two positions with a refined ratio of 0.636 (10): 0.364 (10). In the molecule, the pyridine rings (C1–C5/N1) are approximately planar with the maximum deviations of 0.033 (10) Å at N1A and 0.063 (17) Å at C1B for the major and minor components, respectively. The mean planes of these pyridine rings form dihedral angles of 17 (2)° in the major component and 18 (2)° in the minor component with the respective formamide groups (N2/C6/O1) attached to them. This crystal structure is closely related to that of *N*-[6-(hydroxymethyl)pyridin-2-yl]-2,2-dimethylpropanamide (Goswami, Dey, Chantrapromma *et al.*, 2005).

In the crystal packing (Fig. 2 & Fig. 3), intermolecular N—H···O and C—H···O hydrogen bonds (Table 1) link the molecules into a two-dimensional network parallel to the *ab* plane.

### Experimental

The title compound is synthesized by a known reaction procedure (Yamada & Momose, 1981; Goswami *et al.*, 1989) as follows. In a round-bottomed flask, *N*-(6-bromomethyl-pyridine-2-yl)-2,2-dimethyl propionamide (500 mg, 1.84 mmol) and Co(PPh_3_)_3_Cl (1.76 g, 2 mmol) was kept under nitrogen atmosphere. Dry, degassed benzene (50 ml) was added dropwise to the flask maintaining at 0–15 °C temperature around the flask. The reaction was continued for half an hour. The deep green colour turns blue, an indication of the completion of the reaction. Then benzene was evaporated and the product extracted with CHCl_3_. The solvent was then evaporated and purified by silica gel (100–200 mesh) column chromatography using ethyl acetate and petroleum ether (1:4) as eluent. Single crystals were grown by slow evaporation of a chloroform-methanol (8:2) solution of 1,2-bis(2-pivaloylamino-6-pyridyl)ethane (*m.p.* = 489–491 K, 194 mg, yield = 55%).

### Refinement

All the H atoms were positioned geometrically [C–H = 0.93 to 0.97 Å; N–H = 0.86 Å] and were refined using a riding model, with *U*_iso_(H) = 1.2 *U*_eq_(C, N) or 1.5 *U*_eq_(C). Rigid bond restraint (SAME) was applied to the pyridine ring. The whole molecule is disordered over two positions with a refined ratio of 0.636 (10): 0.364 (10). In the final difference Fourier map, the highest peak and the deepest hole are 0.66 and 0.37 Å from H11D and H11A, respectively.

### Crystal data

**Table:**

C_22_H_30_N_4_O_2_	*F*(000) = 824
*M**_r_* = 382.50	*D*_x_ = 1.163 Mg m^−^^3^
Orthorhombic, *P**b**c**a*	Mo *K*α radiation, λ = 0.71073 Å
Hall symbol: -P 2ac 2ab	Cell parameters from 2706 reflections
*a* = 11.7933 (3) Å	θ = 2.9–20.5°
*b* = 10.3648 (2) Å	µ = 0.08 mm^−^^1^
*c* = 17.8667 (4) Å	*T* = 296 K
*V* = 2183.94 (9) Å^3^	Block, colourless
*Z* = 4	0.36 × 0.15 × 0.10 mm

### Data collection

**Table:**

Bruker SMART APEXII CCD area-detector diffractometer	3221 independent reflections
Radiation source: fine-focus sealed tube	1678 reflections with *I* > 2σ(*I*)
graphite	*R*_int_ = 0.076
φ and ω scans	θ_max_ = 30.1°, θ_min_ = 2.3°
Absorption correction: multi-scan (*SADABS*; Bruker, 2009)	*h* = −13→16
*T*_min_ = 0.973, *T*_max_ = 0.992	*k* = −14→14
36712 measured reflections	*l* = −25→25

### Refinement

**Table:**

Refinement on *F*^2^	Secondary atom site location: difference Fourier map
Least-squares matrix: full	Hydrogen site location: inferred from neighbouring sites
*R*[*F*^2^ > 2σ(*F*^2^)] = 0.062	H-atom parameters constrained
*wR*(*F*^2^) = 0.161	*w* = 1/[σ^2^(*F*_o_^2^) + (0.0587*P*)^2^ + 0.3829*P*] where *P* = (*F*_o_^2^ + 2*F*_c_^2^)/3
*S* = 1.03	(Δ/σ)_max_ < 0.001
3221 reflections	Δρ_max_ = 0.17 e Å^−^^3^
256 parameters	Δρ_min_ = −0.16 e Å^−^^3^
12 restraints	Extinction correction: *SHELXTL* (Sheldrick, 2008), Fc^*^=kFc[1+0.001xFc^2^λ^3^/sin(2θ)]^-1/4^
Primary atom site location: structure-invariant direct methods	Extinction coefficient: 0.0034 (10)

### Special details

**Table:**

Geometry. All e.s.d.'s (except the e.s.d. in the dihedral angle between two l.s. planes) are estimated using the full covariance matrix. The cell e.s.d.'s are taken into account individually in the estimation of e.s.d.'s in distances, angles and torsion angles; correlations between e.s.d.'s in cell parameters are only used when they are defined by crystal symmetry. An approximate (isotropic) treatment of cell e.s.d.'s is used for estimating e.s.d.'s involving l.s. planes.
Refinement. Refinement of *F*^2^ against ALL reflections. The weighted *R*-factor *wR* and goodness of fit *S* are based on *F*^2^, conventional *R*-factors *R* are based on *F*, with *F* set to zero for negative *F*^2^. The threshold expression of *F*^2^ > σ(*F*^2^) is used only for calculating *R*-factors(gt) *etc*. and is not relevant to the choice of reflections for refinement. *R*-factors based on *F*^2^ are statistically about twice as large as those based on *F*, and *R*- factors based on ALL data will be even larger.

### Fractional atomic coordinates and isotropic or equivalent isotropic displacement parameters (Å^2^)

**Table:**

	*x*	*y*	*z*	*U*_iso_*/*U*_eq_	Occ. (<1)
O1A	0.2767 (12)	0.0598 (8)	0.3040 (6)	0.079 (3)	0.636 (10)
N2A	0.2446 (11)	0.2679 (14)	0.3392 (8)	0.0476 (18)	0.636 (10)
H2AB	0.2485	0.3473	0.3255	0.057*	0.636 (10)
N1A	0.1312 (9)	0.3395 (8)	0.4318 (5)	0.067 (3)	0.636 (10)
C1A	0.0598 (10)	0.3242 (10)	0.4903 (5)	0.073 (3)	0.636 (10)
C2A	0.0520 (10)	0.2138 (8)	0.5303 (5)	0.073 (4)	0.636 (10)
H2AA	0.0077	0.2109	0.5734	0.087*	0.636 (10)
C3A	0.1097 (10)	0.1060 (9)	0.5071 (4)	0.0587 (19)	0.636 (10)
H3AA	0.1019	0.0277	0.5320	0.070*	0.636 (10)
C4A	0.1794 (11)	0.1177 (8)	0.4459 (6)	0.055 (3)	0.636 (10)
H4AA	0.2226	0.0480	0.4297	0.066*	0.636 (10)
C5A	0.1845 (7)	0.2338 (8)	0.4088 (4)	0.041 (2)	0.636 (10)
C6A	0.2935 (10)	0.1758 (9)	0.2969 (5)	0.049 (3)	0.636 (10)
C7A	0.3538 (9)	0.2266 (9)	0.2207 (8)	0.052 (3)	0.636 (10)
C8A	0.2636 (6)	0.2620 (9)	0.1715 (3)	0.127 (4)	0.636 (10)
H8AA	0.2196	0.1869	0.1594	0.191*	0.636 (10)
H8AB	0.2945	0.2982	0.1264	0.191*	0.636 (10)
H8AC	0.2160	0.3247	0.1955	0.191*	0.636 (10)
C9A	0.4357 (6)	0.1286 (6)	0.1954 (5)	0.113 (3)	0.636 (10)
H9AA	0.3960	0.0511	0.1820	0.169*	0.636 (10)
H9AB	0.4881	0.1101	0.2351	0.169*	0.636 (10)
H9AC	0.4764	0.1605	0.1528	0.169*	0.636 (10)
C10A	0.4251 (6)	0.3487 (4)	0.2434 (4)	0.0838 (18)	0.636 (10)
H10A	0.4634	0.3820	0.2001	0.126*	0.636 (10)
H10B	0.4800	0.3251	0.2806	0.126*	0.636 (10)
H10C	0.3754	0.4136	0.2632	0.126*	0.636 (10)
C11A	−0.0048 (12)	0.4445 (9)	0.5168 (6)	0.146 (4)	0.636 (10)
H11A	−0.0847	0.4225	0.5164	0.175*	0.636 (10)
H11B	0.0159	0.4583	0.5687	0.175*	0.636 (10)
O1B	0.304 (2)	0.0619 (16)	0.3078 (10)	0.089 (5)	0.364 (10)
N2B	0.257 (2)	0.254 (2)	0.3499 (15)	0.050 (4)	0.364 (10)
H2BB	0.2879	0.3288	0.3476	0.060*	0.364 (10)
N1B	0.1365 (16)	0.3484 (15)	0.4367 (9)	0.066 (5)	0.364 (10)
C1B	0.0801 (16)	0.3399 (14)	0.5017 (7)	0.049 (3)	0.364 (10)
C2B	0.0611 (19)	0.2206 (16)	0.5319 (7)	0.086 (8)	0.364 (10)
H2BA	0.0090	0.2085	0.5704	0.103*	0.364 (10)
C3B	0.122 (2)	0.1198 (19)	0.5029 (11)	0.094 (7)	0.364 (10)
H3BA	0.1199	0.0415	0.5283	0.113*	0.364 (10)
C4B	0.1855 (19)	0.1273 (16)	0.4388 (12)	0.066 (6)	0.364 (10)
H4BA	0.2232	0.0567	0.4184	0.079*	0.364 (10)
C5B	0.1887 (18)	0.2479 (16)	0.4074 (11)	0.064 (5)	0.364 (10)
C6B	0.2962 (18)	0.1758 (18)	0.2901 (10)	0.062 (6)	0.364 (10)
C7B	0.3565 (14)	0.2338 (18)	0.2344 (14)	0.053 (4)	0.364 (10)
C8B	0.4674 (12)	0.272 (3)	0.2505 (7)	0.187 (10)	0.364 (10)
H8BA	0.4664	0.3372	0.2884	0.281*	0.364 (10)
H8BB	0.5022	0.3051	0.2060	0.281*	0.364 (10)
H8BC	0.5097	0.1986	0.2681	0.281*	0.364 (10)
C9B	0.2959 (17)	0.340 (2)	0.1898 (11)	0.210 (12)	0.364 (10)
H9BA	0.2871	0.4152	0.2207	0.314*	0.364 (10)
H9BB	0.2227	0.3098	0.1743	0.314*	0.364 (10)
H9BC	0.3402	0.3617	0.1465	0.314*	0.364 (10)
C10B	0.3652 (19)	0.1244 (13)	0.1673 (7)	0.145 (7)	0.364 (10)
H10D	0.4015	0.1616	0.1243	0.218*	0.364 (10)
H10E	0.2904	0.0961	0.1540	0.218*	0.364 (10)
H10F	0.4088	0.0522	0.1847	0.218*	0.364 (10)
C11B	0.0373 (8)	0.4652 (12)	0.5286 (4)	0.060 (3)	0.364 (10)
H11C	0.1012	0.5200	0.5412	0.072*	0.364 (10)
H11D	−0.0062	0.4514	0.5740	0.072*	0.364 (10)

### Atomic displacement parameters (Å^2^)

**Table:**

	*U*^11^	*U*^22^	*U*^33^	*U*^12^	*U*^13^	*U*^23^
O1A	0.128 (6)	0.036 (3)	0.072 (3)	−0.004 (3)	0.017 (3)	−0.009 (2)
N2A	0.055 (3)	0.041 (4)	0.047 (3)	0.007 (2)	0.015 (3)	0.003 (3)
N1A	0.081 (5)	0.049 (4)	0.070 (5)	−0.001 (3)	0.044 (4)	−0.002 (3)
C1A	0.076 (5)	0.076 (4)	0.065 (5)	0.005 (3)	0.020 (4)	−0.016 (3)
C2A	0.077 (5)	0.071 (6)	0.070 (6)	−0.011 (4)	0.025 (4)	−0.001 (4)
C3A	0.073 (4)	0.060 (3)	0.042 (3)	−0.007 (3)	0.001 (3)	0.008 (2)
C4A	0.074 (5)	0.043 (4)	0.048 (4)	−0.007 (3)	−0.006 (3)	0.016 (3)
C5A	0.042 (3)	0.040 (4)	0.041 (3)	0.008 (3)	0.004 (3)	−0.006 (3)
C6A	0.060 (5)	0.043 (5)	0.044 (3)	−0.003 (3)	0.001 (3)	0.014 (3)
C7A	0.069 (4)	0.046 (3)	0.043 (5)	−0.007 (2)	0.013 (3)	−0.004 (3)
C8A	0.085 (3)	0.235 (10)	0.062 (2)	−0.026 (5)	−0.013 (3)	0.065 (4)
C9A	0.106 (4)	0.073 (3)	0.159 (6)	0.004 (3)	0.073 (4)	−0.025 (3)
C10A	0.094 (4)	0.071 (3)	0.086 (3)	−0.021 (2)	0.025 (3)	0.000 (2)
C11A	0.181 (9)	0.091 (4)	0.165 (8)	0.045 (7)	0.105 (6)	0.014 (6)
O1B	0.129 (11)	0.062 (7)	0.074 (6)	0.041 (6)	0.040 (6)	0.027 (5)
N2B	0.069 (7)	0.025 (3)	0.056 (7)	0.001 (4)	−0.002 (4)	0.010 (4)
N1B	0.085 (9)	0.054 (7)	0.060 (7)	0.018 (5)	−0.008 (6)	−0.015 (5)
C1B	0.061 (5)	0.059 (5)	0.027 (3)	−0.004 (4)	−0.006 (3)	−0.005 (3)
C2B	0.098 (13)	0.129 (18)	0.030 (6)	0.019 (10)	0.008 (6)	0.015 (7)
C3B	0.118 (14)	0.079 (9)	0.086 (10)	−0.020 (8)	0.007 (8)	0.038 (7)
C4B	0.062 (8)	0.084 (13)	0.051 (7)	0.021 (8)	0.014 (5)	−0.013 (8)
C5B	0.071 (9)	0.055 (7)	0.068 (9)	−0.035 (6)	−0.004 (7)	0.013 (6)
C6B	0.059 (9)	0.059 (10)	0.068 (8)	0.028 (7)	0.002 (6)	−0.039 (6)
C7B	0.044 (5)	0.083 (8)	0.032 (6)	0.021 (5)	0.003 (3)	−0.019 (4)
C8B	0.096 (10)	0.37 (3)	0.095 (7)	−0.108 (14)	0.009 (7)	−0.006 (14)
C9B	0.203 (19)	0.23 (2)	0.196 (17)	0.135 (15)	0.127 (15)	0.154 (15)
C10B	0.218 (17)	0.124 (8)	0.094 (7)	−0.057 (11)	0.095 (10)	−0.047 (6)
C11B	0.063 (4)	0.098 (9)	0.019 (2)	0.020 (4)	0.000 (3)	−0.010 (3)

### Geometric parameters (Å, °)

**Table:**

O1A—C6A	1.225 (11)	O1B—C6B	1.23 (2)
N2A—C6A	1.348 (17)	N2B—C5B	1.31 (3)
N2A—C5A	1.473 (14)	N2B—C6B	1.42 (3)
N2A—H2AB	0.8600	N2B—H2BB	0.8600
N1A—C5A	1.328 (7)	N1B—C5B	1.318 (13)
N1A—C1A	1.352 (7)	N1B—C1B	1.341 (12)
C1A—C2A	1.353 (8)	C1B—C2B	1.368 (13)
C1A—C11A	1.536 (13)	C1B—C11B	1.47 (2)
C2A—C3A	1.372 (7)	C2B—C3B	1.368 (13)
C2A—H2AA	0.9300	C2B—H2BA	0.9300
C3A—C4A	1.374 (8)	C3B—C4B	1.372 (13)
C3A—H3AA	0.9300	C3B—H3BA	0.9300
C4A—C5A	1.375 (7)	C4B—C5B	1.372 (13)
C4A—H4AA	0.9300	C4B—H4BA	0.9300
C6A—C7A	1.624 (14)	C6B—C7B	1.36 (3)
C7A—C8A	1.429 (14)	C7B—C8B	1.39 (2)
C7A—C9A	1.473 (12)	C7B—C9B	1.54 (2)
C7A—C10A	1.572 (12)	C7B—C10B	1.65 (2)
C8A—H8AA	0.9600	C8B—H8BA	0.9600
C8A—H8AB	0.9600	C8B—H8BB	0.9600
C8A—H8AC	0.9600	C8B—H8BC	0.9600
C9A—H9AA	0.9600	C9B—H9BA	0.9600
C9A—H9AB	0.9600	C9B—H9BB	0.9600
C9A—H9AC	0.9600	C9B—H9BC	0.9600
C10A—H10A	0.9600	C10B—H10D	0.9600
C10A—H10B	0.9600	C10B—H10E	0.9600
C10A—H10C	0.9600	C10B—H10F	0.9600
C11A—C11A^i^	1.302 (17)	C11B—C11B^i^	1.530 (19)
C11A—H11A	0.9700	C11B—H11C	0.9700
C11A—H11B	0.9700	C11B—H11D	0.9700
			
C6A—N2A—C5A	120.6 (11)	C5B—N2B—C6B	140 (2)
C6A—N2A—H2AB	119.7	C5B—N2B—H2BB	109.9
C5A—N2A—H2AB	119.7	C6B—N2B—H2BB	109.9
C5A—N1A—C1A	116.0 (7)	C5B—N1B—C1B	121.6 (13)
N1A—C1A—C2A	123.4 (8)	N1B—C1B—C2B	118.8 (13)
N1A—C1A—C11A	116.9 (8)	N1B—C1B—C11B	113.3 (11)
C2A—C1A—C11A	119.4 (8)	C2B—C1B—C11B	127.7 (12)
C1A—C2A—C3A	119.7 (8)	C1B—C2B—C3B	117.1 (14)
C1A—C2A—H2AA	120.2	C1B—C2B—H2BA	121.5
C3A—C2A—H2AA	120.2	C3B—C2B—H2BA	121.5
C2A—C3A—C4A	117.8 (8)	C2B—C3B—C4B	124.0 (14)
C2A—C3A—H3AA	121.1	C2B—C3B—H3BA	118.0
C4A—C3A—H3AA	121.1	C4B—C3B—H3BA	118.0
C3A—C4A—C5A	119.1 (7)	C5B—C4B—C3B	114.1 (13)
C3A—C4A—H4AA	120.4	C5B—C4B—H4BA	123.0
C5A—C4A—H4AA	120.4	C3B—C4B—H4BA	123.0
N1A—C5A—C4A	123.5 (6)	N2B—C5B—N1B	124.3 (18)
N1A—C5A—N2A	106.9 (8)	N2B—C5B—C4B	112.4 (17)
C4A—C5A—N2A	129.6 (8)	N1B—C5B—C4B	123.0 (14)
O1A—C6A—N2A	124.5 (10)	O1B—C6B—C7B	125.0 (17)
O1A—C6A—C7A	118.5 (10)	O1B—C6B—N2B	112 (2)
N2A—C6A—C7A	115.3 (9)	C7B—C6B—N2B	118 (2)
C8A—C7A—C9A	118.5 (10)	C6B—C7B—C8B	118 (2)
C8A—C7A—C10A	110.5 (7)	C6B—C7B—C9B	116.9 (15)
C9A—C7A—C10A	106.5 (7)	C8B—C7B—C9B	109.9 (16)
C8A—C7A—C6A	105.8 (8)	C6B—C7B—C10B	105.0 (15)
C9A—C7A—C6A	108.6 (7)	C8B—C7B—C10B	106.5 (14)
C10A—C7A—C6A	106.3 (9)	C9B—C7B—C10B	98.3 (16)
C7A—C8A—H8AA	109.5	C7B—C8B—H8BA	109.5
C7A—C8A—H8AB	109.5	C7B—C8B—H8BB	109.5
H8AA—C8A—H8AB	109.5	H8BA—C8B—H8BB	109.5
C7A—C8A—H8AC	109.5	C7B—C8B—H8BC	109.5
H8AA—C8A—H8AC	109.5	H8BA—C8B—H8BC	109.5
H8AB—C8A—H8AC	109.5	H8BB—C8B—H8BC	109.5
C7A—C9A—H9AA	109.5	C7B—C9B—H9BA	109.5
C7A—C9A—H9AB	109.5	C7B—C9B—H9BB	109.5
H9AA—C9A—H9AB	109.5	H9BA—C9B—H9BB	109.5
C7A—C9A—H9AC	109.5	C7B—C9B—H9BC	109.5
H9AA—C9A—H9AC	109.5	H9BA—C9B—H9BC	109.5
H9AB—C9A—H9AC	109.5	H9BB—C9B—H9BC	109.5
C7A—C10A—H10A	109.5	C7B—C10B—H10D	109.5
C7A—C10A—H10B	109.5	C7B—C10B—H10E	109.5
H10A—C10A—H10B	109.5	H10D—C10B—H10E	109.5
C7A—C10A—H10C	109.5	C7B—C10B—H10F	109.5
H10A—C10A—H10C	109.5	H10D—C10B—H10F	109.5
H10B—C10A—H10C	109.5	H10E—C10B—H10F	109.5
C11A^i^—C11A—C1A	122.2 (9)	C1B—C11B—C11B^i^	113.2 (10)
C11A^i^—C11A—H11A	106.8	C1B—C11B—H11C	108.9
C1A—C11A—H11A	106.8	C11B^i^—C11B—H11C	108.9
C11A^i^—C11A—H11B	106.8	C1B—C11B—H11D	108.9
C1A—C11A—H11B	106.8	C11B^i^—C11B—H11D	108.9
H11A—C11A—H11B	106.6	H11C—C11B—H11D	107.8

Symmetry codes: (i) −*x*, −*y*+1, −*z*+1.

### Hydrogen-bond geometry (Å, °)

**Table:**

*D*—H···*A*	*D*—H	H···*A*	*D*···*A*	*D*—H···*A*
C10A—H10C···O1A^ii^	0.96	2.46	3.409 (12)	171
N2A—H2AB···O1A^ii^	0.86	2.26	3.100 (16)	168

Symmetry codes: (ii) −*x*+1/2, *y*+1/2, *z*.
